# Inheritance of pheromone profiles from aged *D. melanogaster*

**DOI:** 10.17912/micropub.biology.000459

**Published:** 2021-10-11

**Authors:** Samuel G Brown, Dova B Brenman-Suttner, Abigail G McInnes, Katlynn Lew, Amanda J Moehring, Johannes H Bauer, Anne F Simon

**Affiliations:** 1 Department of Chemistry, California State University Sacramento, CA, USA; 2 Current: Department of Biology, York University, Toronto, ON, Canada; 3 Department of Biology, University of Western Ontario, London, ON, Canada

## Abstract

Through aging, *D. melanogaster* males and females change their social spacing. Flies are initially more social, but reduce sociability as they grow older. This preferred social space is inherited in their progeny. Here, we report that in females, the profiles of cuticular hydrocarbons (CHC), which are known to promote social interaction between individuals, similarly are affected by age. Importantly, for a subset of those CHC, the progeny’s CHC levels are comparable to those of their parents, suggesting that parental age influences offspring CHC expression. Those data establish a foundation to identify the relationship between CHC levels and social spacing, and to understand the mechanisms of the inheritance of complex traits.

**Figure 1. Age-dependent CHC patterns may be inherited to the F1 generation:**
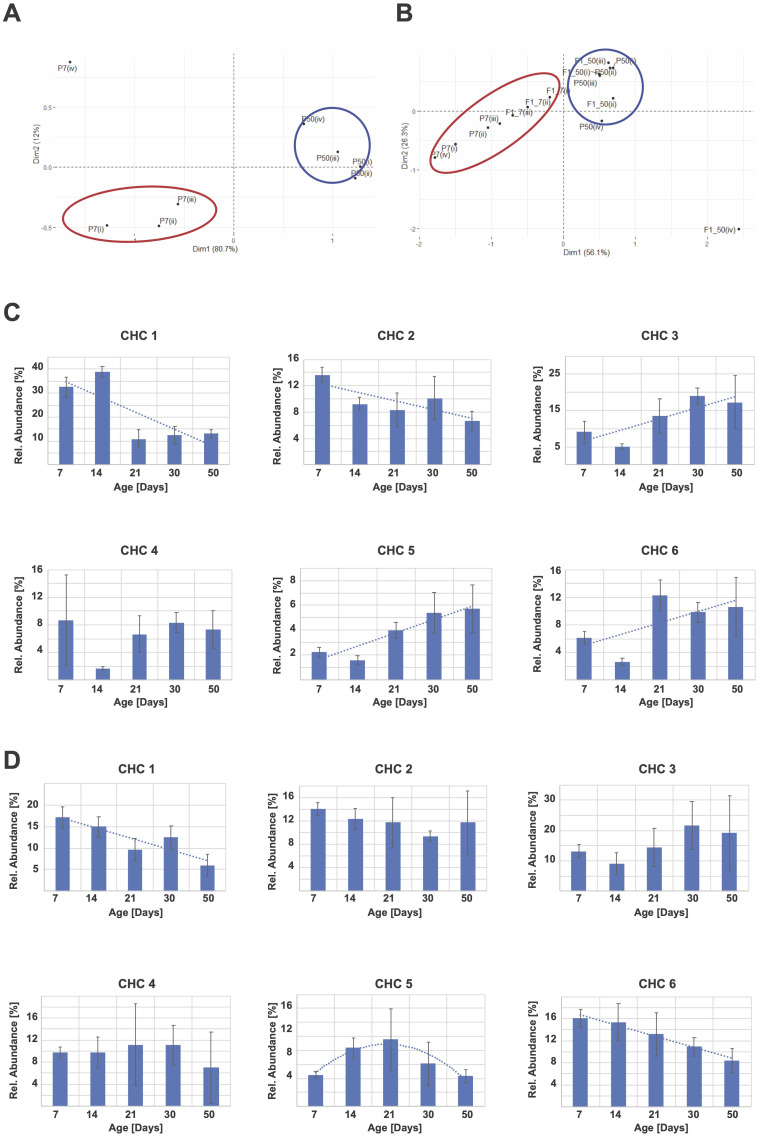
**A)** PCA analysis of CHC amounts of young (7 days old, red circles) and old female flies (50 days old, blue circles) cluster in distinct regions of the PCA graph, suggesting age-dependent changes of CHC expression levels. **B)**CHC amounts in young 7 days old offspring of 50-day-old parents (blue circles, P50 and F1_50) cluster in different PCA graph areas compared to flies of the same age of 7 days , and offspring of young 7 days old parents (red circles, P7 and F1_7). **P**: female parents; **F1_X**: F1 female offspring of the female parents of the indicated ages; roman numerals depict individual replicates. **C)** Age affects CHC expression levels in female flies. Flies of the indicated ages were analyzed for CHC expression. CHC are shown in proportional representation of each CHC peak and depicted as “% of total CHC”. Trendlines are shown as dotted lines for CHC with a of p<0.05 for age correlation. **D)** Age-dependent changes to CHC expression in females may be transmitted to their female offspring. 7-day-old female offspring of parents of the indicated ages were analyzed for CHC profiles. Most CHC are unchanged, regardless of parental age. A few CHC show deviations from day 7 expression levels (best fit trendlines are shown for CHC with a p<0.05 for parental age correlation.

## Description

Pheromones modulate social behaviors like aggregation, aggression, attraction, foraging, group behavior and courtshipin insects (Amrein 2004; Antony *et al.* 1985; Billeter *et al.* 2012; Bontonou and Wicker-Thomas 2014; Fletcher 1968; Greene and Gordon 2003; Krupp *et al.* 2008; Lebreton *et al.* 2012; Lu and Teal 2001; Pankiw 2004; Smedal *et al.* 2009; Wang and Anderson 2010). Molecularly, many pheromones are long-chain hydrocarbons that differ in chain length and saturation status and have recently been proposed to also play a role in the regulation of aging and longevity. For example, the brood pheromone of the honeybee *A. mellifera* has been shown to suppress extreme longevity (Smedal *et al.* 2009), and pheromone sensing is associated with modulation of life span in the roundworm *C. elegans* (Kawano *et al.* 2005; Ludewig *et al.* 2013). In the vinegar fly *Drosophila melanogaster,* most pheromones are produced by specialized cells called oenocytes (for review see Martins and Ramalho-Ortigao 2012) that are associated with the fly fat body. Following synthesis, those fly pheromones are displayed on the cuticle of the animal and referred to as cuticular hydrocarbons (CHC). CHC profiles change with increasing age (Kuo *et al.* 2012), and altering CHC levels in the adult changes both mating behavior and longevity (Joseph *et al.* 2018). In addition, the putative pheromone co-receptor Or83b has been shown to affect fly life span (Libert *et al.* 2007). Finally, male *D. melanogaster* prefer younger females to older females, and this evaluation is largely made through the females age-specific CHC profiles (Kuo *et al.* 2012). Together, these data suggest that pheromone levels may be related to aging and longevity and may thus serve as aging biomarkers (Yew and Chung 2015).

Some health traits, such as onset of diabetes, weight or even longevity, have been suggested to be transmitted to subsequent generations by epigenetic-inheritance mechanisms. Data obtained from an isolated human population in Sweden shows that parental diet influences the longevity of their offspring (Bygren *et al.* 2001; Kaati *et al.* 2007). Furthermore, health traits, especially the development of diabetes, were also transmitted to the next generation (Kaati *et al.* 2002). Data obtained in mice suggests that a paternal low-protein diet leads to epigenetic changes that are transmitted to the offspring (Carone *et al.* 2010), while a maternal high-fat diet leads to increased body size that persists into the F3 generation in mice (Dunn and Bale 2011). Importantly, manipulation of genes involved in epigenetic modification has been shown to change parental longevity in the nematode *C. elegans*, which can be transmitted for several generations (Greer *et al.* 2010). Epigenetic inheritance may affect not just metabolism, but even behavioral traits, such as learning and memory, mating and courtship, and circadian behaviour (for review Shimaji *et al.* 2019), from mice to Drosophila. And in Drosophila, young progeny of old *D. melanogaster* maintain a social spacing behavior that is similar to the behavior of old parents, rather than resembling the behavior of young flies (Brenman-Suttner *et al.* 2018). Together, these data suggest that social behavior may be an age-dependent and epigenetically-inherited trait, with implications for the aging process as well.

Social spacing is the preferred distance between individuals of an undisturbed group, and is a very basic form of social interaction, that probably takes place before more complex behaviors such as aggression or mating. Social spacing is maintained through a balance between attractive and repulsive cues, possibly including, but not limited to, vision but not classical olfaction (Simon *et al.* 2012). How the central nervous system is underlying the decision process of spacing has been investigated: A sex specific neural circuit is emerging as a modulator of social spacing. It involves dopaminergic signalling (Fernandez *et al*. 2017, Xie *et al.* 2018), and cholinergic neurons of the mushroom body (Burg *et al.* 2013). At the synaptic level, Neurobeachin (an anchor protein- Wise *et al.* 2015) and Neuroligin (a cell adhesion protein – Yost *et al.* 2020) are implicated in social space, as well. Finally, FoxP, a highly conserved transcription factor associated to human neurodevelopmental and speech disorders, is also crucial in flies for their control of social spacing (Castells-Nobau *et al.* 2019). However, we do not know which of the perceived cues lead to appropriate social spacing. Because CHC modulate behavior (Wang *et al.* 2008), including group formation, and CHC profiles are themselves altered by social experience (Everaerts *et al.* 2010; Kent *et al.* 2008; Krupp *et al.* 2008; Yew and Chung 2017), it is possible that CHC also play a role in social spacing within a stable group.

As social-spacing is inherited (Brenman-Suttner *et al.* 2018), we hypothesized that some CHC may similarly be affected by epigenetic inheritance. Furthermore, CHC expression profile changes may be preserved in the first generation offspring of old parents (50 days old). If so, inherited effects on CHC production may underlie the observed shift in social spacing. In this study, we measured age-dependent CHC expression of adult *D. melanogaster* throughout their life span. We then compared CHC expression profiles of aging flies to the CHC profiles of their respective young offspring to determine whether F1 CHC profiles were more similar to the profiles of young or aged parents. Here we focused on females, but further work will address male CHC profiles. In this study, we report the results of our first observations on six unidentified CHCs.

We conducted Principal Component Analysis (PCA) of pheromone profiles of young (7 days old) and old flies (50 days old), and their respective offspring to test whether parental age affects CHC expression patterns. Profiles of young and old flies cluster in distinct regions of the PCA analysis, indicating that pheromone expression patterns change with age, as has been reported previously (**Figure 1A**; Kuo *et al.* 2012). Interestingly, when the pheromone profiles of 7 days old offspring of the respectively aged parents are included in the PCA, the distinct clustering is largely retained (**Fig. 1B**). These data suggest that the CHC profile of young F1 offspring of young parents is similar to the profile of young parents, while the profile of young F1 offspring to old parents is likewise similar to the old parental CHC profile. Since all offspring were measured at 7 days of age, these data demonstrate that age-dependent CHC profile changes are inherited to the offspring, presumably via an epigenetic mechanism.

Next, we investigated the nature of the age-dependent CHC profile changes by analyzing six unidentified CHCs present at all ages in all samples (see methods). Depending on the CHC under observation, CHC abundance may not change, increase, or decrease with age (**Fig. 1C**). In order to elucidate further how age-dependent CHC changes may be transmitted, we then analyzed those same CHC in the 7 days old offspring. As expected, most CHCs do not show different expression levels based on the parental age. However, in a small number of cases, F1 CHCs levels are dependent on parental age (**Fig. 1D**), suggesting that age-dependent expression changes are transmitted to the offspring of these aging flies.

We have recently shown that social avoidance behavior may be an epigenetically inherited trait in vinegar flies (Brenman-Suttner *et al.* 2018). Because behaviors are mediated by pheromones, here we investigated whether pheromone expression levels could likewise be epigenetically inherited. Our data show that the expression level of some pheromones depends on parental expression levels, although not for the majority of CHC. Our results provide a possible explanation for previously observed epigenetically inherited behavioral changes in social spacing. This also suggests that other traits associated with these specific CHC, such as specific behaviors or even longevity, could similarly be inherited. Additional studies will be necessary to identify each CHC, and potentially confirm their involvement in social spacing. However, our data provides supporting evidence for the inheritance of complex traits, and a starting point to investigate the molecular mechanisms of this effect.

## Methods

**Aging fly stock:** As reported by Brenman-Suttner *et al.* (2018). In short, ourlaboratory control strain Canton-S *Drosophila melanogaster* was reared in mixed sex groups in bottles over Jazz Mix media (brown sugar, corn meal, yeast, agar, benzoic acid, methyl paraben and propionic acid; Thermo Fisher Scientific, Waltham, MA, USA). All flies were maintained at 25**°**C, 50% humidity with a 12:12 light: dark cycle, lights on at 08:00. New bottles were made bi-weekly when the parents were less than seven days old. After 5 days of laying eggs, existing flies in bottles were removed to prevent new emerging flies cohabitating with their parents. As soon as the flies started emerging, they were collected from the bottles (40 flies/vial, with approximately equal number of males and females, 7 vials/week) under cold anesthesia. Flies were transferred to new media in vials every two-to-three day and were maintained for up to seven weeks.

**Generating the progeny of old parents:** As described above, the old flies were generated through maintaining Canton-S *Drosophila melanogaster* and transferring them to new food every two days. At different ages (7, 14, 21, 30 and 50 days), their progeny were saved and allowed to develop to adulthood (F1: first generation).

**Preparation of flies for CHC extraction:** As described in Pardy *et al.* (2019). In short, 5 *Drosophila melanogaster* females aged 7, 14, 21, 30, and 50 days were collected under cold anesthesia and placed in Eppendorf tubes in a -4**°**C freezer. The 7 days old female progeny of parents aged 7, 14, 21, 30, and 50 days were also collected and frozen in batches of 5 until later use. We collected at least 3 replicates for each sample.

**CHC extraction:** As described in Pardy *et al.* (2019). In short, the three replicates of 5 frozen flies per treatment were washed 100 μl hexane and then the fly body was removed and discarded. Octadecane (C18) and n-hexacosane (C26) were added to the extract as internal standards (10 ng/μl). Samples were analyzed on an Agilent Technologies (Wilmington, USA) 6890 N dual channel gas chromatograph (GC). Hydrogen was used as the carrier gas.

**CHC profile analysis:** Samples were normalized to the internal standard and a 5% threshold was applied to filter out noise. PCA analysis was then performed using the open software suite RStudio (https://www.rstudio.com/; RRID:SCR_000432) and the factoextra package function. The PCA analysis was performed using approximately 37 individual elution peaks, that were present in at least 2 of our biological replicates.

**For analysis of age-dependence** of CHC levels, after removal of peaks inconsistent between replicates, noise-corrected GC peaks were re-normalized to total peak intensity. We then applied exclusion criteria: the peaks we focused on had to be present in each of our biological replicates (16 out of the 37 peaks – which was linked to their abundance), as well at all ages tested. Only 6 peaks fulfilled those criteria, and we analyzed their variation with age as a % of total CHC.

**Statistical Analysis:** Statistical analyses, including One-way ANOVA for CHC age-dependence, were performed using the Prism suite of biostatistical software (GraphPad, San Diego; RRID:SCR_002798).
