# Corrigendum: Inheritance of pheromone profiles from aged *D. melanogaster*

**DOI:** 10.17912/micropub.biology.000597

**Published:** 2022-07-05

## Description

**Two corrections have been made to this article:**

1. A closing parenthesis has been added to the end of the Figure 1D legend text. 

2. Pardy et al. 2021 was cited in the text, however, it was missing from the references. This source has now been added. 

Pardy, J.A., Rundle, H.D., Bernards, M.A., and Moehring, A.J. (2019). The genetic basis of female pheromone differences between *Drosophila melanogaster* and *D. simulans* . Heredity (Edinb) *122* , 93-109. PMID: **29777168**

